# Evaluation of the scatter doses in the direction of the buccal mucosa from dental metals

**DOI:** 10.1120/jacmp.v16i3.5374

**Published:** 2015-05-08

**Authors:** Hiroaki Shimamoto, Iori Sumida, Naoya Kakimoto, Keisuke Marutani, Ryoko Okahata, Ai Usami, Tomomi Tsujimoto, Shumei Murakami, Souhei Furukawa, Sotirios Tetradis

**Affiliations:** ^1^ Department of Oral and Maxillofacial Radiology Osaka University Graduate School of Dentistry Osaka Japan; ^2^ Section of Oral and Maxillofacial Radiology UCLA School of Dentistry Los Angeles CA USA; ^3^ Department of Radiation Oncology Osaka University Graduate School of Medicine Osaka Japan

**Keywords:** radiation therapy, scattered radiation, dental metal, single field, 3D CRT, IMRT

## Abstract

The presence of dental metals creates radiation dose perturbation due to scattered radiation during radiation therapy for the head and neck region. The purpose of our study was to compare the scatter doses resulting from various dental metals in the direction of the buccal mucosa among a single‐field technique, three‐dimensional conformal radiation therapy (3D CRT), and intensity‐modulated radiation therapy (IMRT) during radiation therapy for the head and neck region. We used nine metal cubes with 10 mm sides, which were placed inside a water phantom. The scatter doses from the cubes in the direction of the buccal mucosa were measured using radiochromic films. The films were placed perpendicularly to the surface of the cubes. The phantom was irradiated with a 4 MV photon energy by a linear accelerator for all techniques. In the single‐field technique, the scatter doses from dental metals showed 3.7%–19.3% dose increases, and gold showed the largest dose increase. In 3D CRT, the scatter doses from dental metals showed 1.4%–6.9% dose increases, which were within the measurement uncertainty (except for gold). In IMRT, the scatter doses from dental metals showed only 1.4%–4.3% dose increases, which were all within the measurement uncertainty. During radiation therapy for the head and neck region, the scatter doses from the tested dental metals in the direction of the buccal mucosa in 3D CRT or IMRT were lower than those using the single‐field technique. However, there were no differences between the scatter doses resulting from particular dental metals in the direction of the buccal mucosa in 3D CRT and those in IMRT, except for gold.

PACS number: 87

## I. INTRODUCTION

When metal objects are in the path of megavoltage X‐ray and gamma ray beams, a significant amount of scattered radiation arises.^1^, ^2^, ^3^, ^4^ During radiation therapy for the head and neck region, the presence of dental metals in the oral cavity creates dose perturbation due to scattered radiation in both the upstream and downstream directions at the surface of dental metals. When megavoltage photons interact with high electron density inhomogeneities, such as dental metals located inside water‐ or tissue‐equivalent phantoms, two distinct dose perturbations are observed: 1) an upstream dose increase due to backscatter, which may be the primary source of intraoral mucositis in the surrounding normal tissue in a clinical setting, and 2) a downstream dose decrease due to attenuation over the influence of the dose increase due to forward scatter, which might cause an underprescribed dose being administered to the tumor.^(5)^ The magnitude of the dose increase due to backscatter strongly depends on the atomic number of the scatterer for both photons^1^, ^2^, ^3^, ^4^ and electrons.^6^, ^7^, ^8^, ^9^

Radiation dose perturbation at tissue–titanium interfaces and gold dental fillings in patients with head and neck cancers have been discussed in various studies.^10^, ^11^, ^12^ However, to our knowledge, there have been few previous reports that compared the scatter doses resulting from dental metals among different irradiation methods. Recently, Mail et al.^(13)^ reported that the scatter doses in the direction of the buccal mucosa from a dental metal for single, parallel‐opposed fields and volumetric‐modulated arc therapy (VMAT) were significantly different. However, they evaluated only a single dental metal. We suggest that the evaluation of the scatter doses from various dental metals in the direction of the buccal mucosa among different irradiation methods is needed, as many kinds of metals are used clinically, and the magnitude of scatter doses differs according to their atomic number or composition.

The aim of this study was to compare the scatter doses resulting from various dental metals in the direction of the buccal mucosa, and to also compare these doses among the conventional single‐field technique, three‐dimensional conformal radiation therapy (3D CRT), and intensity‐modulated radiation therapy (IMRT) during radiation therapy for the head and neck region.

## II. MATERIALS AND METHODS

The magnitude of scatter doses from various dental metals was measured using a water phantom. In the present study, we used nine metal cubes measuring 10×10×10 mm, which included gold (Au), silver (Ag), copper (Cu), titanium (Ti), aluminum (Al), silver‐palladium‐gold (AgPdAu) alloy (50% Ag, 20% Pd, 14.4% Cu, 12% Au, 3.6% iridium (Ir)+zinc (Zn)+indium (In)), Ag alloy (72% Ag, 13% Zn, 9% tin (Sn), 6% In), cobalt‐chromium (CoCr) alloy (52% Co, 25% Cr, 14% tungsten (W), 8% gallium (Ga), 1% Al), and nickel‐chromium (NiCr) alloy (78.8% Ni, 19.5% Cr, 1.1% silicon (Si), 0.4% iron (Fe), 0.2% Al), in addition to an acrylic cube with identical dimensions as a control, since the density of the acrylic cube is close to that of water. Each cube was placed inside a cubic water phantom of 20×20×20 cm. Scatter doses from the cubes in the direction of the buccal mucosa were measured by using radiochromic films (GafChromic EBT2, International Specialty Products, Wayne, NJ). EBT2 films from the same lot were used to avoid any intersheet film uncertainties. With regard to the intrasheet film uncertainties, Mizuno et al.^(14)^ reported that the maximum coefficient of variation as evaluated by the net optical density in a single EBT2 film was 3.0% in an early lot, but was no higher than 0.8% in later lots. In this study, we used a later lot.

**Figure 1:**
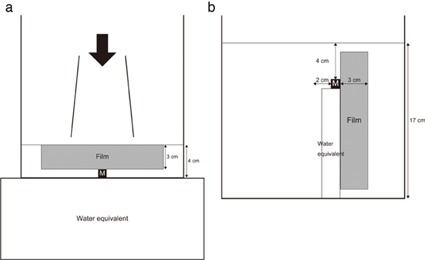
The phantom settings: (a) the single‐field technique; (b) 3D CRT and IMRT. M=metal; Film=Gafchromic EBT2 film; water equivalent=water–equivalent materials.

In the single‐field technique, each test substance cube was set in the center of the acrylic water phantom, as shown in Fig. 1(a). A film of 3 cm width was perpendicularly fixed so that it was in contact with the center of the cube top, parallel to the coronal plane. In other words, the film position was decided on the assumption that the geometry corresponded to the direction of the buccal mucosa in clinical settings.

The test cube was also placed at the isocenter. Water was put in the phantom to a depth of 4 cm to match the surface of the water and the upper edge of the film. Water‐equivalent materials (Tough Water, Kyoto Kagaku, Kyoto, Japan) were placed below the water phantom to fulfill the full scatter condition. The phantom was irradiated with 4 MV photon energy by a linear accelerator (ONCOR Impression Plus, Siemens Medical Solutions, Erlangen, Germany), a radiation dose of 200 monitor units (MUs), a field size of $10\times 10\;{\text{cm}}^{2}$, and the water surface was set to a source–surface distance of 100 cm.

In 3D CRT and IMRT, water was put in the phantom to a depth of 17 cm, as shown in Fig. 1(b). Each test substance cube was placed on water‐equivalent materials (RW3, PTW, Freiburg, Germany) to 4 cm down from the water surface, and was offset 2 cm from the center of the phantom. A 3 cm wide film was perpendicularly fixed so that it was in contact with the center of the cube side, parallel to the coronal plane. The offset position of the cube was selected to parallel the position that corresponded to the left first molar of the mandible in a patient used in the present study.

The phantom was irradiated using a single‐field technique, 3D CRT or IMRT, respectively. After the irradiation, the phantom was scanned using a LightSpeed VCT (GE Healthcare, Milwaukee, WI) under the standard radiation therapy planning protocol; 120 kVp, 220 mA with a reconstructed voxel size of $0.98\times 0.98\times 1.25\;{\text{mm}}^{3}$, and all CT data were imported into the XiO (version 4.50, Elekta, Stockholm, Sweden) treatment planning system. The scatter doses from all CT data obtained using the phantom were calculated using the XiO treatment planning system. The convolution/superposition algorithm with heterogeneity correction was used in the treatment planning. The resolution of the dose calculation was 1×1×1 mm.

**Figure 2:**
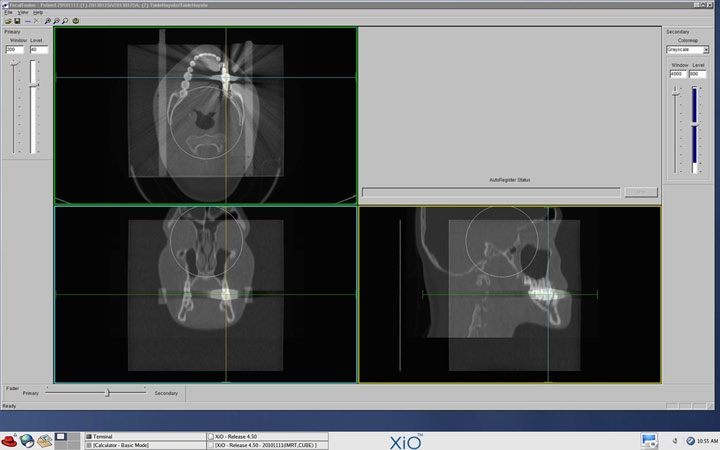
Fusion images between the phantom and the patient. The dental metal of the phantom was located just left of the first molar of the mandible in the patient on the CT images.

**Figure 3:**
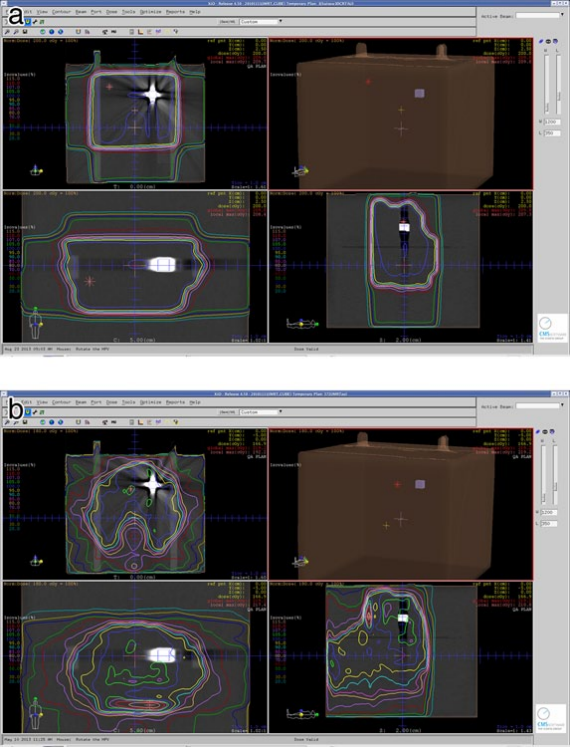
The dose distribution: (a) 3D CRT; (b) IMRT.

In 3D CRT and IMRT, we first delineated the gross tumor volume (GTV) and clinical tumor volume (CTV) using the XiO treatment planning system, using the CT images from a patient who had left side tongue cancer and bilateral lymph node metastases. Second, the 3D CRT plan with the four‐field box technique and IMRT plan with seven directions and 99 segments were generated in the XiO treatment planning system. After that, CT images of the phantom were fused to those of the patient using the position where the test substance cube of the phantom was located just left of the first molar of the mandible in the patient as a reference (Fig. 2). Finally, these plans for the patient were applied to the phantom on the CT images. Figure 3 shows the dose distributions in cases where the treatment plans were applied to the phantom. As shown in Fig. 3, we planned to prescribe 200 cGy to dental metals in 3D CRT and IMRT.

To evaluate the scatter doses from the test substance cubes in the direction representing the buccal mucosa, the upper edge of the film coinciding with the water surface was defined as a depth of 0 mm, and the under edge of the film in contact with the test substance cube was defined as a depth of 30 mm in the single‐field technique. In 3D CRT and IMRT, the film edge being in contact with the test substance cube was defined as a depth of 30 mm and the opposite film edge was defined as a depth of 0 mm.

The films were scanned at 150 dpi with automatic film scanners over 24 hrs after irradiation, and the dose profile data at the position through the center of the cube were obtained. Each scan was performed after a 1 min interval after five warm‐up scans.

Ahead of the measurements of various test substance cubes, the phantom was repeatedly measured 10 times in the presence of Ti in the single‐field technique to evaluate the measurement uncertainty. The measurement uncertainty was defined as 2 SD in pixel‐by‐pixel values at the same position in the dose profile data.

## III. RESULTS

**Figure 4:**
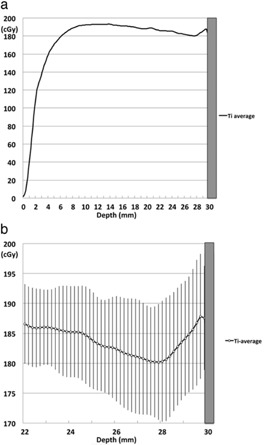
The scatter doses from Ti in the single‐field technique: (a) the average of 10 replicates in the presence of Ti; (b) a magnified view of the areas shown in Fig. 4(a), plus 2 SDs of the 10 replicates of the data with Ti, which was 6.3–10.5 cGy. Ti=titanium.

The mean dose of the 10 repeated measurements with the Ti in the single‐field technique cube is shown in Fig. 4(a). Figure 4(b) shows a magnified view of the Fig. 4(a) ±2 SD range of the 10 repeated measurements, which ranged from 6.3–10.5 cGy (3.4%–5.7% measurement uncertainty).

**Figure 5:**
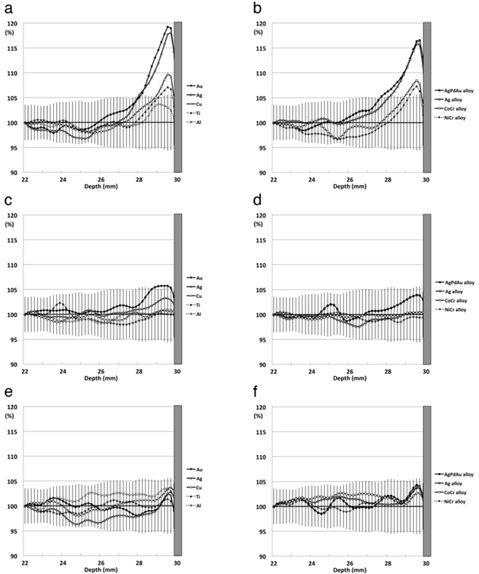
The scatter doses from the dental metals: (a) the single‐field technique in pure metals; (b) the single‐field technique in dental alloys; (c) 3D CRT in pure metals; (d) 3D CRT in dental alloys; (e) IMRT in pure metals; (f) IMRT in dental alloys. All graphs show the percent scatter doses based on the dose found when acrylic was used as a control. Moreover, all data from dental metals are normalized to a depth of 22 mm, where there was no influence of scattered radiation, to correct the variability of measurement doses and compare the scatter doses from the different dental metals. Au=gold;Ag=silver;Cu=copper;Ti=titanium;Al=aluminum;AgPdAu=silver–palladium–gold;CoCr=cobalt–chromium;NiCr=nickel–chromium.

Figure 5 presents all of the data from the various dental metals as the percent scatter doses based on the dose in the experiments using the acrylic cube as a control. All data from dental metals were normalized at a depth of 22 mm, where there was no influence of the scattered radiation, to correct the variability of measurement doses and compare the scatter doses from dental metals.

**Figure 6:**
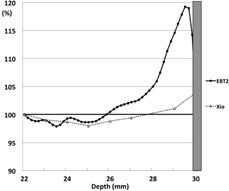
An example of the calculated scatter doses determined using the XiO treatment planning system and the measured scatter doses on the GafChromic EBT2 films in the presence of Au with the single‐field technique. The XiO treatment planning system underestimated the actual scatter doses in the area adjacent to Au.

In the single‐field technique, the scatter doses from dental metals showed 3.7%–19.3% dose increases (Figs. 5(a) and 5(b)). The scatter dose from Au showed the largest dose increase, which was 19.3%, followed by Ag (18.1%), the AgPdAu alloy (16.6%), the Ag alloy (15.8%), Cu (9.8%), the CoCr alloy (8.5%), the NiCr alloy (7.3%), Ti (7.1%), and Al (3.7%). The scatter dose from Al was within the measurement uncertainty. In addition, the maximum dose increases could only be observed within a 2 to 3 mm region upstream from the water–metal interface. The calculated scatter doses from the XiO treatment planning system were lower than the measured scatter doses of GafChromic EBT2 films for all dental metals (Fig. 6).

In 3D CRT, the scatter doses from dental metals showed 1.4%–6.9% dose increases, which were within the measurement uncertainty, except for Au (Figs. 5(c) and 5(d)). In IMRT, the scatter doses from dental metals showed only 1.4%–4.3% dose increases, which were all also within the measurement uncertainty (Figs. 5(e) and 5(f)). The scatter doses from tested dental metals in 3D CRT or IMRT were lower than those from the single‐field technique. The scatter dose from Au in IMRT was less than that in 3D CRT; however, there were no differences between the scatter doses from any of the other metals between 3D CRT and IMRT because they were within the measurement uncertainty.

## IV. DISCUSSION

Dental crowns are most likely to be set on the first molars of jaws because most sites of predilection for dental caries are there, but not on incisors or premolars. Although the materials of dental crowns, posts, and cores may vary between countries, metal dental crowns, metal posts, and metal cores are most commonly used as treatments in Japan as they are covered by the Japanese national health insurance. Hence, we simulated that the test substance cube of the phantom was located just left of the first molar of the mandible in the patient, as shown in Fig. 2. All alloys using in this study are covered by Japanese national health insurance. In the present study, we used five pure metal and four dental alloy metal cubes. As pure metals are not clinically used for dental implants, except for Ti, they might not be strictly described as dental metals. However, measurements of scatter doses from pure metals with specific atomic numbers are valuable to collect baseline values, as well as to evaluate results from previous studies. In addition, some of the dental alloys examined in the present study contained these pure metals. Metal dental crowns are usually made to be 1 mm thick at the maximum. However, most dental crowns are used with metal posts and cores in the clinical setting for the purpose of retaining the tooth strength after teeth are treated with pulpectomy. As a result, almost all tooth crowns are replaced by metal. The size of metal cubes in this study was decided based on the size of a crown of a molar.

Dental restorations and prostheses are routinely made of various kinds of metals which create considerable artifacts on CT images. The metallic artifacts, therefore, introduce a major challenge in the delineation for organs and other structures, and also for ensuring the correct dose distribution in patients with head and neck cancers.^(5)^ Dose perturbations due to high electron density inhomogeneity in dental metals have been measured and calculated in research settings with Monte Carlo (MC) methods.^(15)^ In many of the commercially available treatment planning systems, only the attenuation of the photon beam in the inhomogeneities is considered, whereas the backscattering effect at the interfaces is usually not taken into account.^(16)^ The superposition algorithms, such as the one incorporated in the XiO treatment planning system, although being one of the most accurate and widely used algorithms used in the clinical setting,^(17)^ has been unsuccessful in correctly calculating dose perturbations for high density inhomogeneity.^17^, ^18^ Hence, a detailed analysis of the quantum of backscattered radiation is important for radiation therapy planning which includes high electron density inhomogeneities in the therapeutic radiation field.

In the present study, the calculated scatter doses of the XiO treatment planning system were less than the measured scatter doses on GafChromic EBT2 films. In previous reports, Spirydovich et al.^(5)^ reported that the backscatter doses obtained with the GafChromic EBT films and MC methods were in good agreement with one another. Shimozato et al.^(19)^ reported that the MC data showed the same patterns as the EBT2 data at the surface of dental metals. EBT2 film has a high spatial resolution, low energy dependency, near tissue‐equivalent density $\left(Z^{\text{EBT}2}=6.84\;\text{compared to}\;Z^{\text{water}}=7.42\right)$ and minimal intra‐ and intersheet nonuniformity. In addition, EBT2 film is not affected by repeated scanning, and exhibits postirradiation development similar to that of EBT film, and is not affected by natural light if handled correctly.^20^, ^21^ On the other hand, the calculated scatter doses from metal dental crowns on the XiO or Eclipse (Varian Medical Systems) treatment planning systems were appreciably less than the measured scatter doses on GafChromic EBT2 films or the calculated scatter doses on MC methods, although the calculated scatter doses from metal dental crowns on the XiO were same as those on Eclipse.^(19)^ In the upstream region of an inhomogeneity, the treatment planning systems underestimated the actual scatter doses, as they failed to predict well‐known dose increases near an inhomogeneity due to increased backscatter.^(5)^ In the present study, the GafChromic EBT2 films were scanned at 150 dpi for resolution of the dose measurement of 0.17 mm. In contrast, the resolution of the dose calculation using the XiO treatment planning system was 1 mm. We propose that the low resolution of the dose calculation in the XiO treatment planning system might have cause the underestimation of the scatter doses.

The measurement uncertainty (2 SD) in this phantom study was 3.4%–5.7%. In previous reports, the overall dose uncertainty (2 SD) at 200 cGy in Gafchromic EBT2 film dosimetry was 3.8%–5.6%.^21^, ^22^ The results of our study are, therefore, consistent with these previous reports.

The scattering of doses by pure metals is strongly affected by the atomic number.^1^, ^2^, ^3^, ^4^ In the case of dental alloys, the scattered doses are affected by the effective atomic number.^(19)^ In the single‐field technique, the scattered doses were affected by the atomic number of the pure metals. The results of the dental alloys also showed scatter doses influenced by the composition of the alloys. In the GafChromic EBT2 films measurements, Mail et al.^(13)^ previously reported that the scatter doses from amalgam, which contained 50% mercury (Hg), 25% Ag, 14% Sn, 8% Cu, and 3% other trace metals, included 22% dose increases, and Shimozato et al.^(19)^ reported that the scatter doses from AgPdAu alloy (54% Ag, 20% Pd, 12% Au, 12% Cu, 1% Zn, 1% ruthenium (Ru)), showed approximately 40% dose increases. On the other hand, the scatter dose from Au reported by Lin et al.^(23)^ was approximately 25.0% higher, as indicated in the figure in their report. In the present study, the scatter dose from Au only showed a 19.3% increase at the maximum. It is difficult to make direct comparisons among studies because the conditions of the phantoms were different from each other. Furthermore, the backscatter doses from the metallic inhomogeneities depend on many parameters, such as the photon energy, the width and thickness of the inhomogeneity, the thickness of the medium overlying the interface, the atomic number of the inhomogeneity, the field size, and the angle of the beam.^(24)^

The maximum dose increase could only be observed within a 2 to 3 mm region upstream from the water–metal interface due to the short range of the backscattered electrons in this study. Lin et al.^(23)^ showed that the distance of the effects of scattered doses from dental metals was within a 2 mm region at the maximum, and Reitemeier et al.^(25)^ showed that it was at 3.5 mm at the maximum. The results of our study are consistent with these previous reports. On the other hand, Das and Kahn^(24)^ reported that the distance of the effects of scattered doses from lead (Pb) using 4 MV photon energy was at 8 mm at the maximum. Because the atomic number of Au is lower than that of Pb, the distance of the effects of scattered doses from Au was logically less than 8 mm. As such we considered a depth of 22 mm, which is a distance of 8 mm from dental metals, to be a suitable normalizing position.

In IMRT, the scatter doses from dental metals showed only 1.4%–4.3% dose increases, which were all within the measurement uncertainty in this study. Mail et al.^(13)^ reported that the scatter doses from the amalgam in IMRT decreased to a 9.2% dose increase compared to 22% in the single‐field technique. The cause of this decrease could be attributed to the fact that IMRT enabled multidirectional and multisegment irradiation, that is, not all radiation beams were irradiated on the metal cubes. We propose that the results of 3D CRT, which were all within the measurement uncertainty except for Au, also showed a reasonable scattering dose increase because the four‐field box technique was used in 3D CRT. However, there were no differences between the scatter doses from dental metals between 3D CRT and IMRT, except for Au.

Furthermore, the Mail study reported that the use of a plastic dental mold to spare the oral mucosa reduced the backscatter doses from 12.06% to 2.7% in VMAT, as was the case with the single‐field technique in head and neck patients. However, the scatter doses from dental metals in the present study showed only 4.3% dose increases at a maximum in IMRT. Therefore, it might not always be necessary to use a dental mold in IMRT.

There are some limitations associated with the present study. First, we did not measure the scatter doses in the presence of teeth and bones. Although Mail et al.^(13)^ reported that the scatter doses from a single tooth in VMAT showed a 1.6% dose increase compared to that under the toothless condition, greater dose increases would be expected in patients, because there are ample biological structures, but also various amounts and kinds of dental metals. However, it is difficult to unambiguously interpret the data regarding the scatter doses from dental metals in such a complicated situation. Therefore, we performed this phantom study to collect fundamental data regarding the scatter doses from different dental metals and using different techniques. It is fair to say that only few studies have directly evaluated the scatter doses from dental metals in IMRT, and therefore further investigations are needed. Second, we only measured the scatter doses from dental metals using 4 MV photon energy (opposed to the 6 MV typically used in radiation therapy for the head and neck region). Some researchers have reported that the scatter doses from metals using 6 MV were higher than those at 4 MV.^24^, ^26^ According to Gibbs et al.,^(26)^ the data for 6 MV were about 5% higher than those of 4 MV in single and parallel‐opposed fields. We plan to investigate the scatter doses from dental metals using different photon energies in future studies. Third, for 3D CRT, we used a four‐field box technique, although this is not commonly used in radiation therapy for the head and neck region. The reason for this approach was that we could not prescribe 200 cGy to dental metals due to the shape of the phantom, which is different from that of a patient's head and neck (despite the verification of irradiation techniques which were commonly used in 3D CRT for the head and neck region). In the future, we will need to investigate scatter doses from dental metals using a phantom with a shape similar to that of the head and neck. Fourth, for the single‐field technique, we used a different phantom setting from that used in 3D CRT and IMRT. Clinically, the single‐field technique is typically not used except for a preoperation radiotherapy, unlike 3D CRT or IMRT. We first measured the scattered doses from dental metals for the single‐field technique from above as a basic study. Then we used the phantom setting under the assumption that it was closer to the clinical setting. In the single‐field technique, the scatter doses from dental metals showed 3.7%–19.3% dose increases when all beams were irradiated toward the dental metals. We attempted to show that the scatter doses from dental metals in 3D CRT and IMRT were lower than those using the single‐field technique, because 3D CRT enabled multidirectional irradiation, and IMRT enabled multidirectional and multisegment irradiation; that is, not all radiation beams were irradiated onto the metal cubes.

## V. CONCLUSIONS

The scatter doses from tested dental metals in the direction of the buccal mucosa in 3D CRT or IMRT were lower than those in the single‐field technique during radiation therapy for the head and neck region. However, there were no differences between the scatter doses resulting from particular dental metals in the direction of the buccal mucosa in 3D CRT and those in IMRT, except for Au.
